# Fluoride exposure and duration and quality of sleep in a Canadian population-based sample

**DOI:** 10.1186/s12940-021-00700-7

**Published:** 2021-02-18

**Authors:** Jasmyn E. A. Cunningham, Hugh McCague, Ashley J. Malin, David Flora, Christine Till

**Affiliations:** 1 Michael G. DeGroote School of Medicine, McMaster University, Hamilton, Ontario Canada; 2 Institute for Social Research, York University, Toronto, Ontario Canada; 3 Department of Environmental Medicine and Public Health, Icahn School of Medicine at Mount Sinai, New York, NY USA; 4 Faculty of Health, York University, Toronto, Ontario Canada

**Keywords:** Fluoride, Sleep, Canada, Adolescent, Adult, Drinking water

## Abstract

**Background:**

Fluoride from dietary and environmental sources may concentrate in calcium-containing regions of the body such as the pineal gland. The pineal gland synthesizes melatonin, a hormone that regulates the sleep-wake cycle. We examined associations between fluoride exposure and sleep outcomes among older adolescents and adults in Canada.

**Methods:**

We used population-based data from Cycle 3 (2012–2013) of the Canadian Health Measures Survey. Participants were aged 16 to 79 years and 32% lived in communities supplied with fluoridated municipal water. Urinary fluoride concentrations were measured in spot samples and adjusted for specific gravity (UF_SG_; *n* = 1303) and water fluoride concentrations were measured in tap water samples among those who reported drinking tap water (*n* = 1016). We used multinomial and ordered logistic regression analyses (using both unweighted and survey-weighted data) to examine associations of fluoride exposure with self-reported sleep outcomes, including sleep duration, frequency of sleep problems, and daytime sleepiness. Covariates included age, sex, ethnicity, body mass index, chronic health conditions, and household income.

**Results:**

Median (IQR) UF_SG_ concentration was 0.67 (0.63) mg/L. Median (IQR) water fluoride concentration was 0.58 (0.27) mg/L among participants living in communities supplied with fluoridated municipal water and 0.01 (0.06) mg/L among those living in non-fluoridated communities. A 0.5 mg/L higher water fluoride level was associated with 34% higher relative risk of reporting sleeping less than the recommended duration for age [unweighted: RRR = 1.34, 95% CI: 1.03, 1.73; *p* = .026]; the relative risk was higher, though less precise, using survey-weighted data [RRR = 1.96, 95% CI: 0.99, 3.87; *p* = .05]. UF_SG_ was not significantly associated with sleep duration. Water fluoride and UF_SG_ concentration were not significantly associated with frequency of sleep problems or daytime sleepiness.

**Conclusions:**

Fluoride exposure may contribute to sleeping less than the recommended duration among older adolescents and adults in Canada.

**Supplementary Information:**

The online version contains supplementary material available at 10.1186/s12940-021-00700-7.

## Introduction

Inorganic fluoride can naturally occur in the environment or be introduced through industrial processes or fluoride supplementation programs. A primary method of fluoride supplementation is community water fluoridation, which refers to the practice of adding fluoridation chemicals to drinking water for the purpose of controlling dental caries. Ingestion of fluoridated drinking water is a major source of fluoride intake [1, 2].

As of 2017, approximately 39% of Canadians received fluoridated water via public water systems, with the highest fluoridation rates being in Ontario and Manitoba [3]. At appropriate levels, fluoride in drinking water has consistently been shown to be associated with reduced dental caries in children [1, 4], reducing tooth decay by approximately 30 to 40% [5]. Excess fluoride intake in early life has been associated with enamel fluorosis [4], though increased risk of neurodevelopmental toxicity has been recently reported in populations exposed to optimal fluoride levels [6, 7]. Currently, the maximum acceptable concentration (MAC) of fluoride in drinking water in Canada is 1.5 mg/L, with an optimal water fluoride target of 0.7 mg/L to maximize dental benefits while minimizing fluorosis [8].

In 2006, the National Research Council (NRC) conducted a comprehensive review of the health effects of fluoride exposure [9]. One conclusion was that fluoride is likely to affect pineal functioning, and may cause a decrease in melatonin production [9]. The pineal gland is a small neuroendocrine organ situated near the center of the brain. It sits outside of the blood-brain barrier, and thus the passage of fluoride is not restricted as it is in other areas of the brain. Its tissue is subject to mineralization, with calcification producing concretions up to several millimeters in diameter [10]. This calcification consists of hydroxyapatite, similar to that of bones or teeth [11–13]. It has been found to accumulate high levels of fluoride [10] even from low fluoride consumption due to fluoride’s high affinity for hydroxyapatite [12]. This vulnerability could increase the risk of pineal gland fluoride toxicity [13, 14]. In older individuals, fluoride measurements in the pineal gland have been shown to be roughly equivalent to those in teeth [10].

The pineal gland’s primary function is to synthesize melatonin during the dark portion of the day-night cycle to help maintain normal sleep and circadian rhythms. Melatonin is suppressed by light to the retina and its secretion is controlled by the circadian timing system driven by the circadian pacemaker, the suprachiasmatic nucleus [15]. Given fluoride’s propensity to accumulate in the pineal gland [10, 12], along with the well-established relationships of the degree of pineal gland calcification with human melatonin levels [16–18] and disruption to various sleep-related outcomes (including REM sleep percentage, total sleep time, sleep efficiency, daytime tiredness, and sleep disturbance) [18, 19], further research is needed examining the potential for fluoride to impact sleep outcomes. To date, only one study has investigated the association between fluoride exposure and sleep [20]; none have examined this association in adult humans.

Our study examined the association between fluoride exposure and sleep outcomes in a large Canadian sample using cross-sectional data from Cycle 3 (2012–2013) of the Canadian Health Measures Survey (CHMS). Specifically, we assessed the associations of fluoride concentrations in household tap water and urinary spot samples with self-reported sleep outcomes, including sleep duration and frequency of sleep problems as well as daytime sleepiness. We hypothesized that greater fluoride exposure would be associated with lower-than-optimal sleep duration and an increase in sleeping problems and daytime sleepiness.

## Methods

### Study participants

This study used data from Cycle 3 (2012–2013) of the Canadian Health Measures Survey (CHMS). The CHMS randomly selected individuals ages 3 to 79 years living in Canada, excluding individuals living on reserves and Aboriginal settlements, full-time members of the Canadian Forces, institutionalized individuals, and individuals who were otherwise remote-dwelling or who lived in the northern territories. In total, 5785 respondents from 16 sites across 10 provinces were enrolled in the survey; 2671 provided urine samples (out of the approximately 2950 who were asked to do so) and 2188 provided tap water samples that were analyzed for fluoride (for more information, please see [21]) [22]. For the current study, we selected individuals who were between 16 and 79 years and had a fluoride measurement in either a tap water or urine sample (*n* = 1396) (see Additional File 1 for a participant flowchart). We included only participants over the age of 15 years because parents are less likely to set the bedtime of older adolescents [23] and we were interested in measures of sleep duration that reflect habits of participants themselves rather than sleep durations artificially imposed via parenting.

Among those with complete covariate and sleep outcome data, there were 1016 participants with fluoride concentrations measured in their tap water and 1303 participants who had urinary fluoride concentration measurements. The urinary fluoride sample (*n* = 1303) included individuals regardless of whether they drink tap water whereas the tap water sample (*n* = 1016) only included individuals if they reported drinking tap water. Of the urinary fluoride sample, 387 (29.7%) lived in regions without community water fluoridation, 415 (31.9%) lived in regions with community water fluoridation, and 501 (38.5%) lived in regions with unreported or mixed community water fluoridation (i.e. due to unclear site boundaries, only some or intermittent municipal water fluoridation). For a description of the determination of water fluoridation status in the CHMS sample, see [24].

### Fluoride measures

Fluoride concentrations in urine spot samples (non-fasting; non-standard collection time; first-catch urine) were diluted with an ionic adjustment buffer and analyzed using an Orion pH meter with a fluoride ion selective electrode. Analyses of urine samples were completed at the Laboratoire de santé publique du Québec under standardized operating procedures [25]. For quality assurance and control protocols, see [25]. The limit of detection (LoD) for urinary fluoride was 10 μg/L [25]; all of the urinary fluoride values were above this LoD. Concentrations of urinary fluoride were adjusted for specific gravity (UF_SG_; mg/L) rather than creatinine because urinary creatinine concentrations can demonstrate more variation with factors such as age, sex, ethnicity/race, body mass index (BMI), and time of day of sample collection [26–28].

Tap water samples (mg/L) were collected at participants’ homes and were analyzed for fluoride by the Laboratoire de santé publique du Québec [25] using a basic anion exchange chromatography procedure with a LoD of 6 μg/L. Values below the LoD were replaced with an imputed value of LoD/√2 [29].

### Sleep measures

Self-reported habitual sleep duration was based on participants’ response to the question: *How many hours do you usually spend sleeping in a 24 h period, excluding time spent resting?* Values were recorded to the nearest half hour, with a minimum value of 1 and a maximum value of 24. Based on these values, participants were then categorized into one of three categories: lower than recommended sleep duration, recommended sleep duration, or higher than recommended sleep duration. Categories were based on the National Sleep Foundation’s sleep range recommendations [30], which corresponded to age groups as follows: 8–10 h for teenagers aged 16 to 17 years, 7–9 h for adults aged 18 to 64, and 7–8 h for adults aged 65 or older.

Frequency of sleep problems and frequency of daytime sleepiness were also ascertained by questionnaire. Participants responded to the questions: *How often do you have trouble going to sleep or staying asleep?* and *How often do you find it difficult to stay awake during your normal waking hours when you want to?* Both questions were answered on a five-point scale, with responses categorized as *never, rarely, sometimes, most of the time,* or *all of the time.*

### Covariates

We selected covariates a priori that had theoretical relevance to sleep outcomes or fluoride exposure [31–36]. These covariates included BMI, ethnicity (white or non-white), total household income (log transformed), chronic health condition (yes/no), sex, and age. Examples of chronic health conditions included a heart condition, multiple sclerosis, kidney dysfunction, and use of a colostomy bag. We identified confounders by using a directed acyclic graph (DAG) (see Additional File 2).

### Statistical analysis

All analyses were completed with Stata (version 15) at the Research Data Centres (RDC) at York University and McMaster University. Descriptive statistics were obtained for all exposure variables, outcome variables, and covariates. The current study reports non-weighted descriptive data because results were first released from the RDC using non-weighted data (and we are not permitted to release both weighted and non-weighted descriptive data). Subsequent analyses applied survey weights and 500 bootstrap weights to permit generalization of the findings to the entire Canadian population. Thus, we report our effect estimates using both unweighted and survey-weighted data.

Spearman correlation was used to test the relationship between UF_SG_ and water fluoride concentration collapsing across age. Multinomial logistic regression was used to model sleep duration as a function of urinary or tap water fluoride, adjusting for covariates. Ordered logistic regression was used to model frequency of sleep problems and daytime sleepiness as a function of urinary or tap water fluoride, adjusting for covariates. Variance inflation factors indicated no concerns with multicollinearity in any of our models. We tested potential departures from linear associations between fluoride exposure and the sleep outcome logits by fitting models with quadratic terms (i.e. UF_SG_^2^). We did not detect non-log-linearity of the effect estimates. We explored potentially influential cases using Cook’s distance and identified a small number of cases with high water fluoride levels ranging between 2.0 to 2.5 mg/L and UF_SG_ concentration values that were above 4 mg/L. Analyses retained the high water fluoride values because they are plausible outcomes (due to some regions in Canada having naturally occurring fluoride levels that exceed the MAC level) and were not deemed influential using Cook’s distance. However, we removed the small number of cases with extreme UF_SG_ values > 4 mg/L from our primary models because there was less certainty that these values were plausible exposure values (exact number of cases not reported due to Statistics Canada confidentiality requirements). While these values are biologically plausible, they are more likely to represent an acute fluoride ingestion (e.g. swallowing toothpaste prior to urine sample) rather than a reliable exposure measure.

We tested potential interactions between fluoride exposure and sex, age, BMI, and ethnicity, respectively; if non-significant, the interaction term was removed. Given the wide age range, we also tested a quadratic interaction effect with age (i.e., fluoride*age^2^). A two-tailed alpha of 0.05 was used as the threshold for statistical significance for all main effects as well as interactions given the large number of interactions tested and their exploratory nature. To aid interpretation, we adjusted the regression coefficients so that they represent the predicted difference in sleep outcome per 0.5 mg/L of fluoride in tap water or urine; 0.5 mg/L corresponds to the approximate difference between mean water or UF_SG_ level among individuals living in fluoridated versus non-fluoridated regions in Canada [24]. We also present regression coefficients representing the predicted difference in sleep outcome per IQR mg/L of fluoride in tap water or urine. Finally, we conducted the following sensitivity analyses: 1) we restricted the sample to adults aged 18 and up; 2) we re-ran the models with the UF_SG_ values > 4 mg/L included.

## Results

Tables 1 and 2 present descriptive statistics (unweighted) for demographic characteristics, fluoride variables, and sleep outcomes for participants included in the current study (see Additional File 1 for demographic characteristics of participants excluded because a water fluoride or urinary fluoride outcome was not collected). Urinary fluoride levels were moderately associated with water fluoride levels (Spearman correlation = 0.45, *p* < .001). Among the 1303 participants with urinary fluoride measurements, 858 (66%) reported sleeping for a duration within the recommended range [30], 377 (29%) reported sleeping less than the recommended duration and 68 (5%) reported sleeping more. Most of the sample (combined total of 689; 53%) reported *never* or *rarely* having trouble going to sleep or staying asleep, with 308 (24%) reporting trouble *most* or *all of the time*. Similarly, most of the sample (*n* = 822; 63%) reported *never* or *rarely* having difficulty staying awake during normal waking hours, with only 93 (7%) reporting difficulty *most* or *all of the time*.

**Table 1:** Demographic characteristics and sleep outcomes according to urinary fluoride and water fluoride samples (unweighted data shown^a^)

	Urinary fluoride sample (*n* = 1303)	Water fluoride sample (*n* = 1016)
*Demographic characteristics*		
Age (years); mean (SD)	42.4 (19.0)	43.3 (18.6)
Total household income (Canadian dollars); Mean (SD)	83,868 (65,489)	87,293 (66,421)
Ethnicity; n (%)		
White	1052 (80.7)	812 (79.9)
Non-white	251 (19.3)	204 (20.1)
Sex; n (%)		
Female	669 (51.3)	515 (50.7)
Male	634 (48.7)	501 (49.3)
BMI category; n (%)		
Underweight	39 (3.0)	30 (3)^b^
Normal weight	507 (38.9)	417 (41)^b^
Overweight	434 (33.3)	345 (34)^b^
Obese	323 (24.8)	224 (22)^b^
Water fluoridation status; n (%)		
Not fluoridated	387 (29.7)	309 (30.4)
Fluoridated	415 (31.9)	342 (33.7)
Missing or mixed fluoridation	501 (38.5)	365 (35.9)
*Sleep outcomes*		
Sleep duration; n (%)		
Lower than recommended	377 (28.9)	290 (28.5)
Within recommended^c^	858 (65.9)	677 (66.6)
Higher than recommended	68 (5.2)	49 (4.8)
Trouble sleeping; n (%)		
Never	389 (29.9)	317 (31.2)
Rarely	300 (23.0)	246 (24.2)
Sometimes	306 (23.5)	217 (21.4)
Most of the time	187 (14.4)	143 (14.1)
All of the time	121 (9.3)	93 (9.2)
Trouble staying awake; n (%)		
Never	439 (33.7)	344 (33.9)
Rarely	383 (29.4)	313 (30.8)
Sometimes	388 (29.8)	291 (28.6)
Most of the time	73 (5.6)	68 (6.7)^d^
All of the time	20 (1.5)	^d^

^a^As directed by Statistics Canada, we are not permitted to report both non-weighted and population-weighted descriptive data. Abbreviations: *BMI* body mass index, *SD* standard deviation. ^b^Approximate sample sizes and rounded percentages reported due to confidentiality requirements by Statistics Canada. ^c^Categories based on the National Sleep Foundation’s recommendations [30]: 8–10 h if ages 16 to 17 years, 7–9 h if ages 18 to 64, 7–8 h if ages 65 or older. ^d^Categories *Most of the time* and *All of the time* were collapsed due to confidentiality requirements by Statistics Canada (value reflects number of participants reporting either response)

**Table 2:** Descriptive statistics for fluoride measures (unweighted data shown)

	N	Mean	SD	25th percentile	50th percentile	75th percentile
Urinary fluoride, adjusted for specific gravity (mg/L)	1303	0.86	0.62	0.46	0.67	1.08
Tap water fluoride (mg/L)	1016	0.24	0.26	0.06	0.12	0.44
Fluoridated	342	0.52	0.22	0.42	0.58	0.69
Non-fluoridated	309	0.05	0.15	0.01	0.01	0.07
Mixed or unknown	365	0.14	0.11	0.09	0.11	0.14

Multinomial logistic regression of sleep duration and water fluoride levels adjusted for covariates showed a significant association between higher water fluoride and a lower sleep duration; specifically, for every 0.5 mg/L higher water fluoride concentration, there was a 34% increased relative risk of reporting sleeping less than the recommended duration (RRR = 1.34; 95% CI: 1.03, 1.73; *p* = .026; Table 3). Water fluoride levels were not significantly associated with sleeping more than the recommended amount, frequency of trouble sleeping, or frequency of daytime sleepiness. Urinary fluoride levels were not significantly associated with any of the sleep outcomes (Table 4), except a significant age by UF_SG_ interaction in the ordinal logistic regression described next. The pattern of results remained consistent using the weighted data (see Additional File 3, Supplemental Table 2 for the weighted coefficients). The weighted multinomial logistic regression model showed that higher water fluoride levels were associated with a higher, though less precise, relative risk of reporting sleeping less than the recommended duration (RRR = 1.96; 95% CI: 0.99, 3.87; *p* = .05). No other associations were found with water fluoride levels and sleep outcomes or with UF_SG_ and sleep outcomes using weighted models.

**Table 3:** Adjusted associations between water fluoride concentration and sleep measures (*n* = 1016)

Outcome	Association (95% CI) for change in outcome per:		*p*
	*0.5 mg/L*	*IQR (0.38 mg/L)*	
Sleep Duration^a^			
Less than recommended	1.34 (1.03, 1.73)	1.25 (1.03, 1.51)	.03
Recommended (ref)	–	–	
More than recommended	0.95 (0.53, 1.71)	0.96 (0.61, 1.50)	.86
Trouble Sleeping^b^	0.95 (0.77, 1.18)	0.97 (0.82, 1.13)	.67
Daytime Sleepiness^b^	1.16 (0.94, 1.44)	1.12 (0.95, 1.32)	.17

^a^Relative risk ratios (RRR)

^b^Odds Ratio (OR)

**Table 4:** Adjusted associations between specific-gravity adjusted urinary fluoride concentration and sleep measures (*n* = 1303)

Outcome	Association (95% CI) for change in outcome per:		
	*0.5 mg/L*	*IQR (0.62 mg/L)*	*p*
Sleep Duration^a^			
Less than recommended	1.02 (0.93, 1.13)	1.02 (0.94, 1.10)	.64
Recommended (ref)	–	–	
More than recommended	0.91 (0.74, 1.13)	0.93 (0.80, 1.10)	.40
Trouble Sleeping^b^	0.96 (0.89, 1.04)	0.97 (0.91, 1.03)	.37
Daytime Sleepiness^b^	1.00 (0.92, 1.08)	1.00 (0.94, 1.06)	.95

^a^Relative risk ratios (RRR)

^b^Odds Ratio (OR)

In the ordinal logistic regression, age modified the association between UF_SG_ and frequency of sleep difficulties (*p* interaction = .046). The nature of the interaction was such that the predicted UF_SG_ association decreased as age increased. Specifically, for a hypothetical participant at the first age quartile (i.e., 26 years), the predicted OR was 1.17, indicating that the odds of reporting a higher level of sleep difficulties was greater with higher levels of UF_SG_ among younger participants; for a participant at the median age (i.e., 40 years), the predicted OR was 1.03, indicating a weak positive association between UF_SG_ and the likelihood of increased sleep difficulties; for a hypothetical participant at the third age quartile (i.e., 60 years), the predicted OR was 0.85, indicating that higher levels of UF_SG_ were associated with a lower likelihood of increased sleep difficulties. There were no other significant interactions between age and UF_SG_ (interaction for sleep duration *p* = .25; interaction for daytime sleepiness *p* = .10) or age and water fluoride concentration (interaction for under-sleeping *p* = .72 and oversleeping *p* = .78; interaction for sleep difficulties *p* = .73; interaction for daytime sleepiness *p* = .95). There were no significant interactions between sex and fluoride variables, ethnicity and fluoride variables, and BMI and fluoride variables (*p* > 0.05 in all cases).

The findings did not change appreciably when we restricted the water fluoride and UF_SG_ samples to people aged 18 and up (Supplemental Tables 3 and 4). Likewise, the results remained consistent when we included the UF_SG_ values > 4 mg/L in predicting sleep outcomes (Supplemental Table 4).

## Discussion

We investigated the association between fluoride exposure and several sleep outcomes in a large population-based sample of older adolescents and adults, using Cycle 3 (2012–2013) of the Canadian Health Measures Survey. As of 2017, approximately 39% of Canadians had access to fluoridated water [3]; in our sample, approximately 32% lived in regions with community water fluoridation, 30% lived in regions without water fluoridation, and 38% lived in areas of mixed community water fluoridation or had missing data.

We found that for every 0.5 mg/L higher water fluoride concentration, there is a 34% increased relative risk of reporting sleeping less than the recommended amount. This result suggests that higher fluoride exposure is associated with sleep deficits in older adolescents and adults. The association between higher water fluoride concentration and sleeping less remained consistent if we restrict the sample to those aged 18 and older (RRR = 1.32). Using weighted data, we found an even stronger, though less precise, association between water fluoride concentration and sleeping less (RRR = 1.96). Among individuals aged 16 and older, we found that approximately 29% of individuals reported sleeping less than the recommended duration. This proportion of individuals reporting low habitual sleep durations is consistent with a larger sample of 10,976 adult respondents of the CHMS of which 32% reported sleeping less than the recommended duration [36]. Insufficient sleep is associated with various adverse outcomes, including changes in mood [37–40], cognition [37, 40–42], and reaction time [40, 43, 44], higher rates of motor vehicle accidents [45], hypertension, cardiovascular disease [46–48], and diabetes [47].

While the direction of causality cannot be discerned due to the cross-sectional nature of the survey, we can provide some hypotheses to explain the finding. Given the tendency of fluoride to accumulate in the pineal gland, the association between fluoride exposure and shorter sleep duration may be explained by an effect on melatonin production and subsequently the timing of sleep. In humans, the pineal gland is subject to calcification and forms concretions. This calcification varies between individuals, but generally increases with age [9, 49]. Sympathetic nervous system innervation forms the major communication between the pineal gland and the superior cervical ganglion [9, 50] and may also serve to maintain these concretions [49]. Additionally, these concretions may reflect prior biosynthetic metabolic activity of the gland, resulting from dark exposure, for example [49]. Fluoride accumulates in the pineal gland to a similar degree as in teeth [10] and the pineal gland in older individuals contains more fluoride than any other soft tissue [49]. This accumulation is likely due to fluoride’s high affinity for hydroxyapatite [12], as pineal fluoride concentration is directly correlated with pineal calcium concentration [10, 49], as well as the fact that it sits outside of the blood brain barrier, has a substantial blood supply [13], and may be ‘sampling’ the blood in circulation [49]. One study found that the association between pineal fluoride and calcium was very strong (*r*^2^ = 0.92) only for pineal glands with high levels of fluoride, which implies that high pineal fluoride is associated with increased calcification [12, 13]. The deposition of fluoride in calcified tissues, such as the pineal gland, bones, and teeth, may represent a defense mechanism against potential fluoride toxicity (in other tissues), which may begin in the prenatal period [13].

Fluoride deposition in the pineal gland and its calcification would most likely exert effects on sleep via changes to pinealocytes and subsequently melatonin output. The pineal gland is composed primarily of pinealocytes, which synthesize melatonin [13, 50]. Pinealocyte calcification is not directly correlated with decreased plasma melatonin; however, it is associated with a decreased number of pinealocytes [13]. Therefore, pinealocyte calcification likely has indirect effects on melatonin biosynthesis, as the degree of *un*calcified pineal tissue is associated with plasma and saliva melatonin [13, 16, 17]. Calcification of the pineal gland may also lead to decreased levels of melatonin in the cerebrospinal fluid [13]. Fluoride has been shown to affect pinealocytes and melatonin in animal models; for example, adult rats showed a 73% increase in these cells after 8 weeks of a fluoride-free diet compared to rats consuming standard fluoridated food and water [14], and a doctoral dissertation showed that prepubescent gerbils receiving a diet high in fluoride had lower melatonin production than those receiving a low-fluoride diet [49]. To our knowledge, there are no studies in living humans on the direct effects of fluoride exposure on the pineal gland or melatonin production and secretion.

These changes in calcification and melatonin excretion are a credible means through which fluoride could impact sleep and circadian outcomes. Calcification of the pineal gland has been shown to be associated directly with sleep complaints, including daytime tiredness and sleep disturbance [19], as well as decreased REM sleep percentage, total sleep time, and sleep efficiency on polysomnographic measurement [18]. Higher uncalcified pineal volume has been associated with less sleep rhythm disturbance, even in otherwise ‘good sleepers’ [16]. Changes in melatonin onset time, peak time, and peak concentration have been associated with sleep disturbances, specifically sleep efficiency [51], and a delay in endogenous melatonin rhythms compared to normal individuals is what typically leads to the clinical manifestation of delayed sleep phase syndrome (DSPS). For individuals with DSPS, exogenous melatonin administration can be an effective means of ameliorating symptoms, by advancing sleep onset and wake times [52]. Alternatively, it is possible that fluoride could directly inhibit enzymes required for melatonin synthesis [13]. Together, these findings imply that a smaller or more calcified pineal gland and subsequent decreased melatonin output could be a risk factor for circadian instability and/or sleep and sleep rhythm disturbances.

Another possible mechanism through which fluoride could exert effects on the central nervous system, including sleep outcomes, is through oxidative stress. Fluoride can be a source of oxidative stress and may induce generation of reactive oxygen species (ROS) and lipid peroxidization [13, 53, 54]. Melatonin and other proteins from the pineal gland act as antioxidants [54]. Therefore, as the pineal gland calcifies and there is decreased melatonin output, there may be increased activity of these ROS and subsequent neural damage [13]. Further research should be conducted to elucidate this potential pathway. Alternatively, mechanisms outside of the pineal gland may be responsible for our findings; for example, hypothyroidism [55, 56] or hypertension [57, 58] could potentially influence the relationship between fluoride exposure and short sleep duration. These health conditions could possibly be on the causal pathway that mediates the association between fluoride exposure and sleep duration.

As far as we are aware, this is only the second human study investigating the effects of fluoride exposure on sleep outcomes, and the first study with an adult sample. Fluoride exposure has been associated with later bed and wake times in 16-to-19 year olds [20]; if participants went to bed later due to a delayed onset of nighttime sleepiness, but were constrained from a later wake time due to conflicting commitments (work, school, etc.), this situation could contribute to a shortened sleep duration as seen in the present study. However, given that our sample included mainly adults (and only adults when we restricted the sample to those aged 18 and older), this generalization from results found in older adolescents should be made cautiously. Additionally, the present study did not include a measure of sleep timing, so we were unable to test this hypothesis. Future studies should continue to investigate effects of fluoride exposure on sleep timing in adults.

Although we found an association between water fluoride and sleep duration, we did not find any associations between measures of fluoride exposure and our other sleep outcomes (frequency of daytime sleepiness or sleep problems). There are several potential reasons for these findings. It is possible that fluoride primarily leads to a disturbance in melatonin secretion in the form of a delay, as outlined above, or to a direct shortening of total sleep time, either of which could manifest as a shortened sleep duration but without accompanying daytime sleepiness or nighttime sleep disturbance. Alternatively, our measurements of sleep outcomes were rudimentary (comprised of a single question capturing each sleep outcome), and it is possible that they were not sensitive enough to capture various sleep pathologies. For example, the question assessing daytime sleepiness asks about difficulty staying awake; participants may have experienced more moderate daytime sleepiness, which would not necessarily have been captured in our data. Similarly, participants would likely not have been aware of disturbances in their sleep architecture (i.e. sleep problems), and our dataset would therefore not include this information. Future research should investigate the relationship between fluoride exposure and sleep using both objective (e.g. polysomnography, actigraphy, dim light melatonin onset) and validated subjective measures (e.g. Pittsburgh Sleep Quality Index [59], Stanford Sleepiness Scale [60]) to better characterize the nature of any sleep and circadian disturbances.

One limitation of this study is that we were unable to account for the presence of sleep disorders or other behaviors that could impact sleep (e.g. exercise, diet, use of melatonin supplements, sleeping pills, or medications). Future studies should investigate the relationship between fluoride exposure, sleep, and these factors; the relationship with physical activity is of particular interest given that a pilot study found that vigorous exercise in adults is associated with a reduction in renal fluoride clearance and an increased trend in plasma fluoride concentration [61]. This could potentially lead to greater uptake by body tissue [62], though this study of mice found no link between chronic exercise and bone fluoride levels. While we did not account for physical activity in our study, we did include BMI as a covariate, which is associated with physical activity levels [63]. Additionally, because our study is cross-sectional, the direction of the association between fluoride exposure and sleep outcomes is not established. Given these limitations and the limited evidence on the topic to date, the results of our study should be viewed as hypothesis-generating.

A strength of our study is that we included two measurements of fluoride exposure: tap water fluoride and specific-gravity adjusted urinary fluoride levels. Our results demonstrate that while water fluoride level was significantly associated with a lower than recommended sleep duration, UF_SG_ was not. This finding is consistent with two other population-based fluoride studies [20, 24], both of which found that water fluoride levels but not fluoride biomarkers (i.e. plasma, urine) were associated with adverse health outcomes. Consistent with their explanation, it is likely that water fluoride measurements act as a proxy for chronic or historical fluoride exposure (assuming the individual’s residence in a community with water fluoridation remains stable), whereas single urinary spot samples represent short-term (contemporaneous) exposure that may fluctuate between subsequent measurements [20]. In terms of risk to pineal gland calcification, fluoride may need time to accumulate in the pineal gland before exerting an impact on sleep. Although it is known that pineal gland calcification increases with age [9, 49], it is not clear whether lifetime exposure drives this relationship. One report [49] suggested that pineal fluoride does not reflect cumulative fluoride exposure of the individual due to a lack of correlation between fluoride in the pineal gland and in bone ash. However, this finding was potentially confounded by other methodological factors, such as the inclusion of bone samples from different types of bone, which have been shown to contain differing levels of fluoride [9, 10, 49]. Moreover, we would not necessarily expect pineal and bone fluoride levels to be correlated given that fluoride is reversibly bound to bone and can be re-released into plasma [64]. Prospective studies are needed to investigate the potential impacts of cumulative fluoride exposure across varying stages of development on sleep outcomes.

## Conclusions

Higher water fluoride concentration was significantly associated with increased risk of reporting fewer than the recommended hours of sleep. This finding suggests that fluoride exposure may contribute to clinically meaningful reductions in sleep duration among individuals living in areas with optimal water fluoridation. These findings should be interpreted in the context of the demonstrated benefits of fluoride exposure for dental health [1, 4, 5, 8, 65].

## Supplementary Information

**Additional file 1.** Participant selection

**Additional file 2.** Directed Acyclic Graph (DAG)

**Additional file 3.** Supplemental Tables

## Data Availability

The data from the Canadian Health Measures Survey (CHMS) Cycle 3 (2012–2013) analyzed during the current study are publicly available here: https://www.statcan.gc.ca/eng/statistical-programs/document/5071_D5_T9_V1

## Abbreviations

BMI: Body Mass Index
CHMS: Canadian Health Measures Survey
CI: Confidence Interval
DSPS: Delayed Sleep Phase Syndrome
IQR: Interquartile Range
LoD: Limit of Detection
MAC: Maximum Acceptable Concentration
NRC: National Research Council
RDC: Research Data Centre
ROS: Reactive Oxygen Species
RRR: Relative Risk Ratio
UF_SG_: Urinary Fluoride adjusted for Specific Gravity
