# Supplementary figures and images for: Fluoride exposure and duration and quality of sleep in a Canadian population-based sample
Source: Environ Health. 2021 Feb 18;20:16. doi: 10.1186/s12940-021-00700-7 (PMC7893939; doi:10.1186/s12940-021-00700-7)

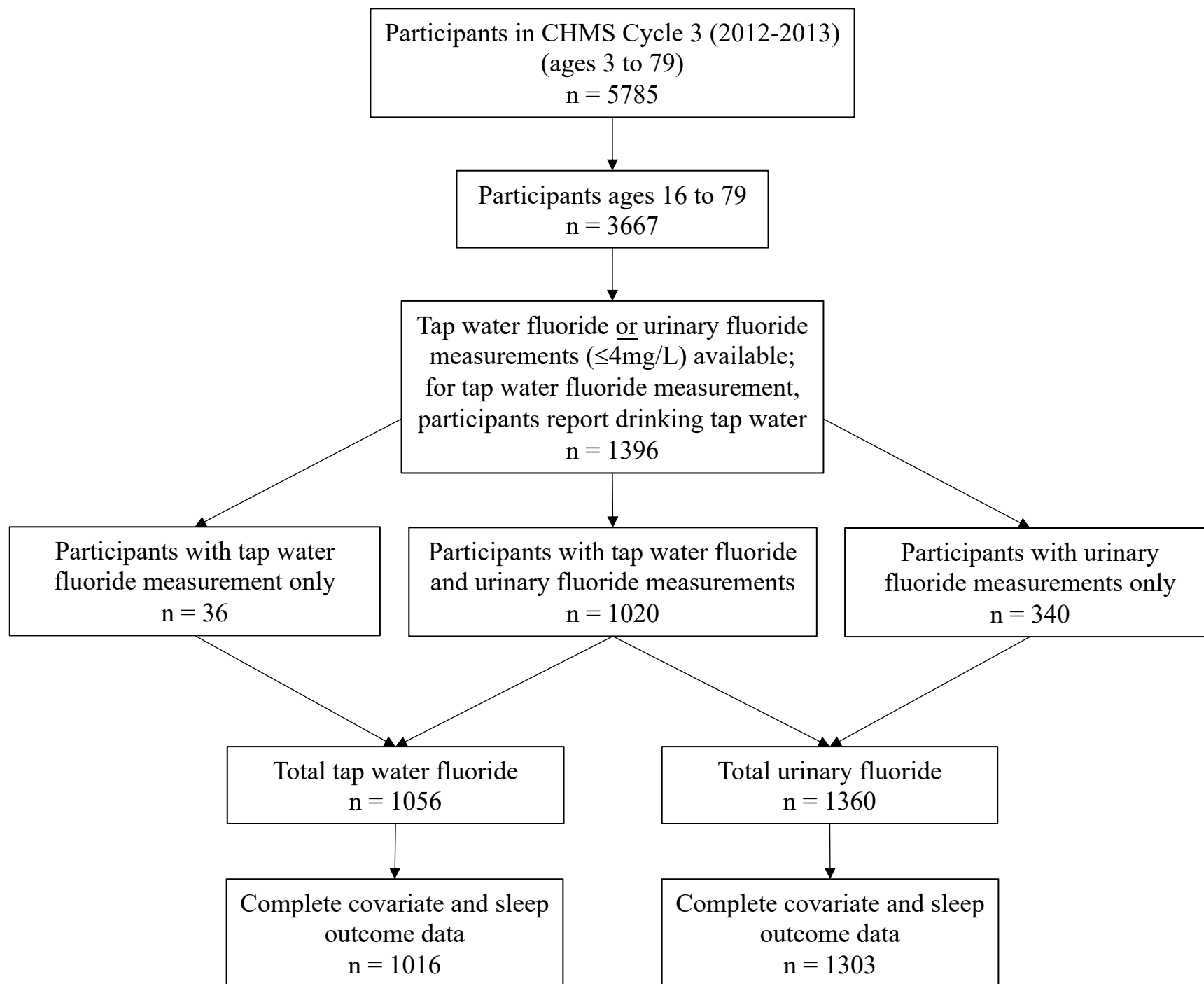

Supplement: Supplementary file 1 — Additional file 1. Participant selection [file 12940_2021_700_MOESM1_ESM.pdf]

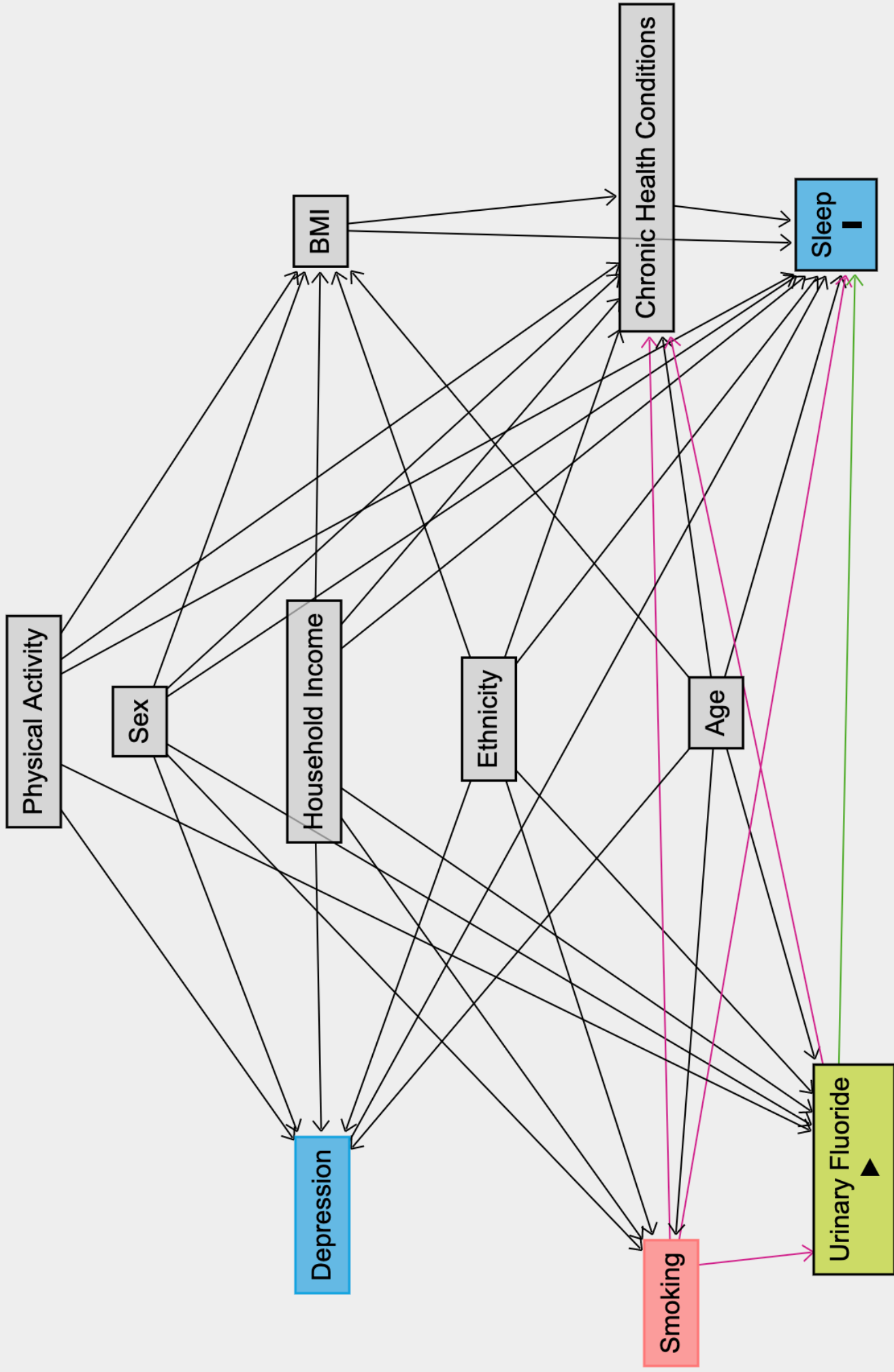

Supplement: Supplementary file 2 — Additional file 2. Directed Acyclic Graph (DAG) [file 12940_2021_700_MOESM2_ESM.pdf]
