# Supplementary material for: Fluoride exposure and duration and quality of sleep in a Canadian population-based sample
Source: Environ Health. 2021 Feb 18;20:16. doi: 10.1186/s12940-021-00700-7 (PMC7893939; doi:10.1186/s12940-021-00700-7)
Supplement: Supplementary file 3 — Additional file 3. Supplemental Tables [file 12940_2021_700_MOESM3_ESM.docx]

Supplemental Table 1. Demographic characteristics and sleep outcomes of the excluded sample (aged 16 years and older).

|  | Excluded Sample (n = 2304) | |
| --- | --- | --- |
| *Demographic characteristics* |  | |
| Age (years); mean (SD) | 46.5 (17.4) | |
| Total household income  (Canadian dollars); Mean (SD) | 77,537 (61,008) | |
| Ethnicity; n (%) |  | |
| White | 1783 (79.6) | |
| Non-white | 456 (20.4) | |
| Sex; n (%) |  | |
| Female | 1157 (50.2) | |
| Male | 1147 (49.8) | |
| BMI category; n (%) |  | |
| Underweight | 42 (1.8) | |
| Normal weight | 810 (35.2) | |
| Overweight | 814 (35.3) | |
| Obese | 638 (28.0) | |
| Water fluoridation status; n (%) |  | |
| Not fluoridated | 709 (30.8) | |
| Fluoridated | 748 (32.5) | |
| Missing or mixed fluoridation | 847 (36.8) | |
| *Sleep outcomes* |  | |
| Sleep duration; n (%) |  | |
| Lower than recommended | 741 (32.2) | |
| Within recommended^+^ | 1410 (61.2) | |
| Higher than recommended | 153 (6.6) | |
| Trouble sleeping; n (%) |  | |
| Never | 646 (28.1) | |
| Rarely | 493 (21.4) | |
| Sometimes | 592 (25.7) | |
| Most of the time | 321 (13.9) | |
| All of the time | 251 (10.9) | |
| Trouble staying awake; n (%) |  | |
| Never | 868 (37.7) | |
| Rarely | 667 (29.0) | |
| Sometimes | 608 (26.4) | |
| Most of the time | 136 (5.9) | |
| All of the time | 22 (1.0) |  |

Abbreviations: BMI = body mass index; SD = standard deviation.  ^+^Categories based on the National Sleep Foundation’s recommendations (30): 8-10 hours if ages 16 to 17 years, 7-9 hours if ages 18 to 64, 7-8 hours if ages 65 or older.

Supplemental Table 2. Adjusted associations between water fluoride concentration and UF_SG_ concentrations and sleep measures using population weighted data.

| Outcome | Estimates (95% CI) | | | |
| --- | --- | --- | --- | --- |
|  | *Water fluoride sample*  n = 1,016  N = 6,412,524 | *p* | *UF_SG_ sample*  n = 1,303  N = 8,217,396 | *p* |
| Sleep Duration^†^ |  |  |  |  |
| Less than recommended | 1.96 (0.99, 3.87) | .05 | 1.12 (0.93, 1.35) | .25 |
| Recommended (ref) | - |  | - |  |
| More than recommended | 0.68 (0.07, 6.67) | .74 | 0.88 (0.56, 1.40) | .60 |
| Trouble Sleeping^#^ | 0.97 (0.45, 2.08) | .94 | 1.47 (0.92, 2.33) | .10 |
| Daytime Sleepiness^#^ | 1.03 (0.68, 1.56) | .90 | 1.32 (0.81, 2.14) | .27 |

*Note:* Effect estimates reflect the change in outcome for each increase in 0.5 mg/L in urinary fluoride or water fluoride concentration.

^†^Relative risk ratios (RRR)

^#^Odds Ratio (OR)

Supplemental Table 3. Adjusted associations between water fluoride concentration and sleep measures for participants aged 18 and older (n = 902).

| Outcome | Estimates (95% CI) | *p* |  |
| --- | --- | --- | --- |
| Sleep Duration^†^ |  |  |  |
| Less than recommended | 1.32 (1.01, 1.73) | .045 |  |
| Recommended (ref) | - |  |  |
| More than recommended | 0.98 (0.54, 1.77) | .95 |  |
| Trouble Sleeping^#^ | 0.96 (0.77, 1.20) | .74 |  |
| Daytime Sleepiness^#^ | 1.13 (0.90, 1.42) | .28 |  |

*Note:* Effect estimates reflect the change in outcome for each increase in 0.5 mg/L in urinary fluoride concentration.

^†^Relative risk ratios (RRR)

^#^Odds Ratio (OR)

Supplemental Table 4. Adjusted associations between specific-gravity adjusted urinary fluoride concentration and sleep measures for sample aged 18 years and older including UF_SG_ values that are higher than 4 mg/L (n > 1140).

| Outcome | Estimates (95% CI) | *p* |  |
| --- | --- | --- | --- |
| Sleep Duration^†^ |  |  |  |
| Less than recommended | 1.03 (0.95, 1.12) | .47 |  |
| Recommended (ref) | - |  |  |
| More than recommended | 0.91 (0.75, 1.11) | .37 |  |
| Trouble Sleeping^#^ | 0.99 (0.93, 1.07) | .88 |  |
| Daytime Sleepiness^#^ | 0.97 (0.90, 1.04) | .41 |  |

Note: Exact number of participants included in this analysis cannot be reported due to Statistics Canada reporting requirements. Effect estimates reflect the change in outcome for each increase in 0.5 mg/L in urinary fluoride concentration.

^†^Relative risk ratios (RRR)

^#^Odds Ratio (OR)
